# Supplementary material for: Assessing the feasibility of the Virtual Reality Education and Acceptance Protocol among baseball and softball players
Source: PLoS One. 2025 Nov 25;20(11):e0337537. doi: 10.1371/journal.pone.0337537 (PMC12646411; doi:10.1371/journal.pone.0337537)
Supplement: S1 File — (DOCX) [file pone.0337537.s001.docx]

**Virtual Reality Education and Acceptance Protocol**

**Stage 1: VR Education**

VR education includes a presentation with images and video that educates participants on the basic functions of VR and its potential uses in their domain (e.g., sport, medicine, etc.) so that they begin with a baseline of understanding. Previous research has demonstrated a positive relationship between educational training and technology adoption [1]. In a sport context, previous research has also shown that athletes might be more willing to use VR if they understand how it can be applied to their sport and if elite athletes in their sport endorse or use it [2], which contributes to perceived usefulness in line with the Technology Acceptance Model [TAM; 3]. As such, the content of the presentation should include domain-specific examples and evidence that individuals with perceived importance to the user has used VR in their shared domain. The presentation lasts approximately 8-10 minutes. Once the presentation is complete, the facilitator(s) shows the users to their respective usage spaces and assists with putting on the VR headsets in preparation for Stage 2.

Note: See the presentation below as an example of Stage 1 with baseball and softball players.

**Stage 2: Acclimation**

This stage consists of two acclimation periods, each lasting approximately 10-15 minutes with 10-12 minutes in between periods for recovery. This structure is based on two factors:

1. Studies have shown reduced VR cybersickness in 15-minute exposures [4, 5].
2. Based on previous research, 10-12 minutes between acclimation periods should provide ample time for recovery if cybersickness occurs [5, 6].

Each acclimation period consists of immersion in a game or application within the VR headset with progressing cognitive workloads. The first application is TriptoVR, an app that allows the user to explore various locations around the world using video recorded with a 360-degree camera. Users complete the Tour of Venice feature (or another location if available), which lasts approximately eight and a half minutes. During this time, they remain seated in a chair. The video includes minimal movement with no tactile involvement. This allows the user to begin to acclimate to the visual component of VR with limited cognitive workload. The second application is First Steps, an application that is provided by Meta on all Quest VR headsets. The app is designed as a tutorial to acclimate users to the virtual experience, and it increases the cognitive workload as the user progresses through the application, including kinesthetic and tactile interactions with the environment. This period lasts approximately 15 minutes.

During recovery periods between uses, users can stand or sit wherever they choose. They can also partake in the crackers and water provided in case of cybersickness. During the recovery time, users may informally discuss their thoughts and experiences with the other users and facilitator(s). During this time, the facilitator(s) preps the next application so that users can begin using it immediately in the next stage.

**Stage 3: Application**

Users are provided an opportunity to apply VR to their specific domain, thus increasing their perceived usefulness, which has been demonstrated as a predictor of intent to use [7]. Participants in this stage are given up to 15 minutes of free time to use a domain-specific application or interact with a domain-specific 360-degree video. In this the user or practitioner implementing the protocol should choose an application that has demonstrated effectiveness for its domain or use a recorded 360-degree video tailored to the user’s needs. For example, when using the VREAP with baseball and softball players, user could be given up to 15 minutes of free time in Win Reality, which is a baseball and softball-specific application designed to train pitch recognition and decision-making. In this stage, users may discontinue use any time during the 15-minute period if they so choose.

**Example of VR Education (i.e., Stage 1) with baseball and softball players**


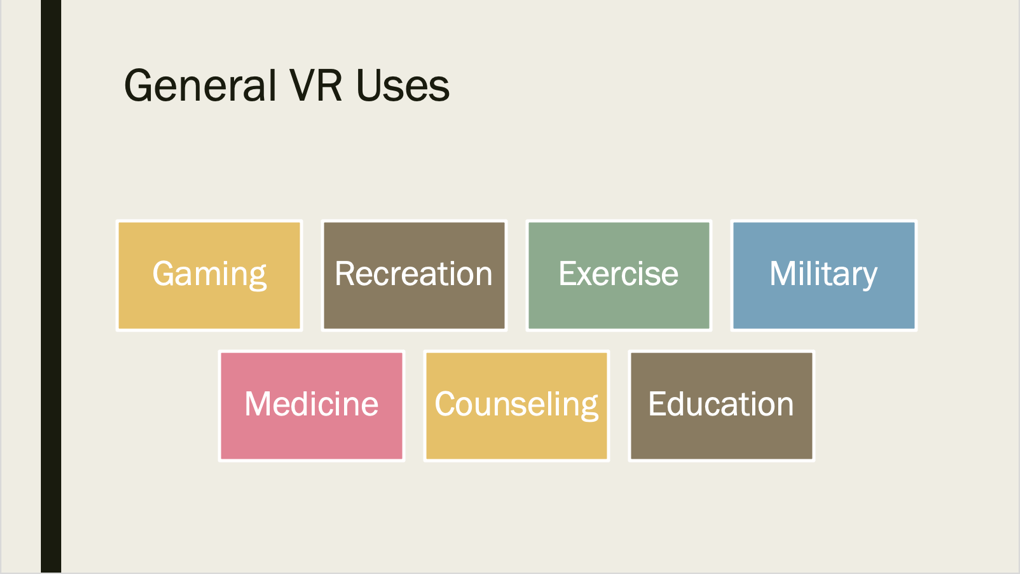

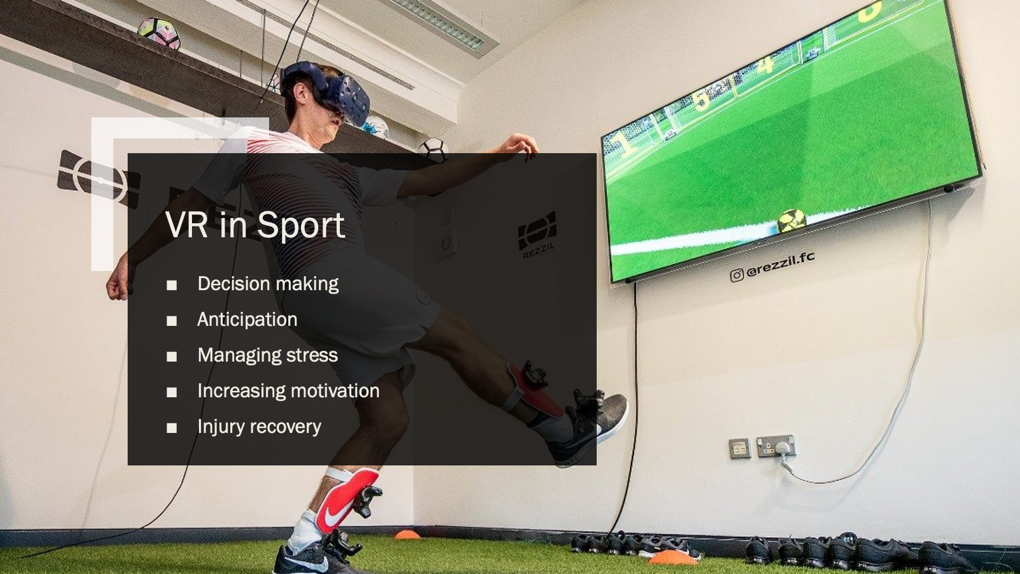

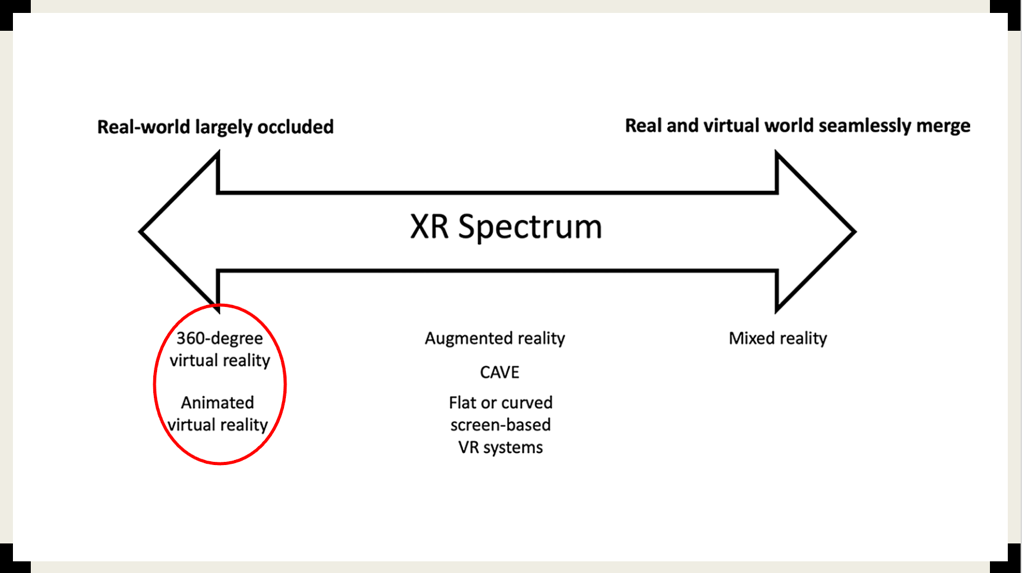

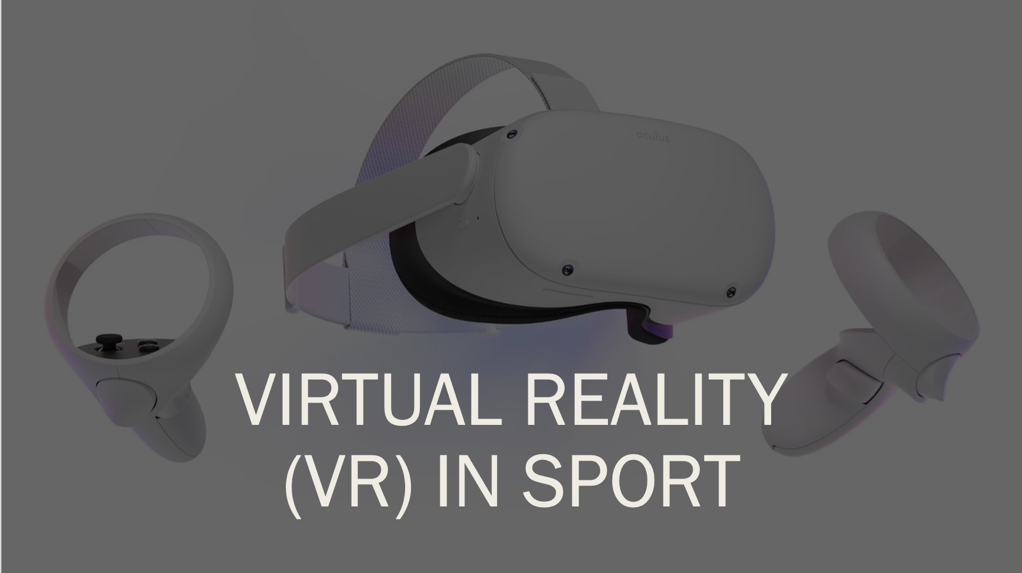


4

3

2

1


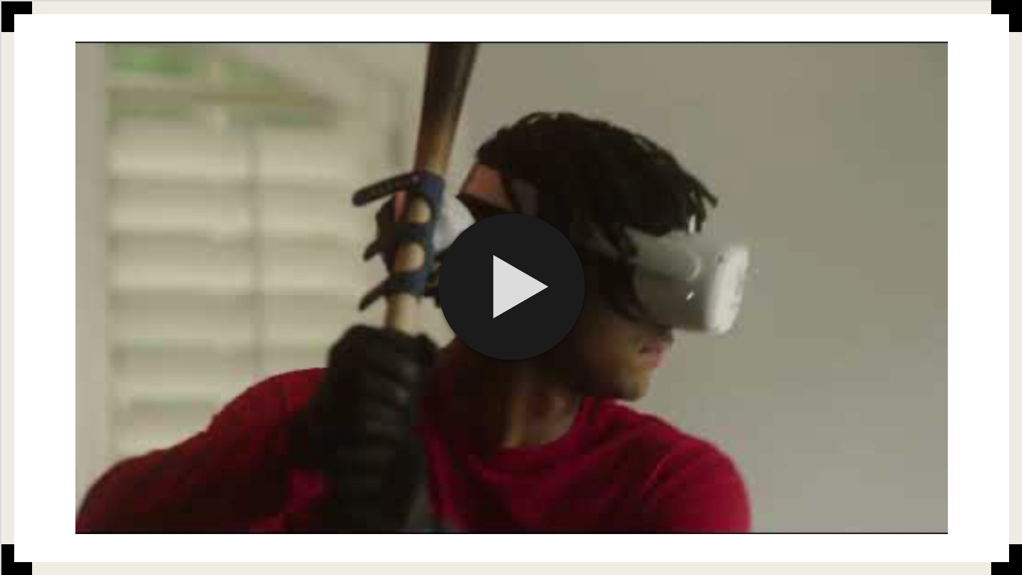

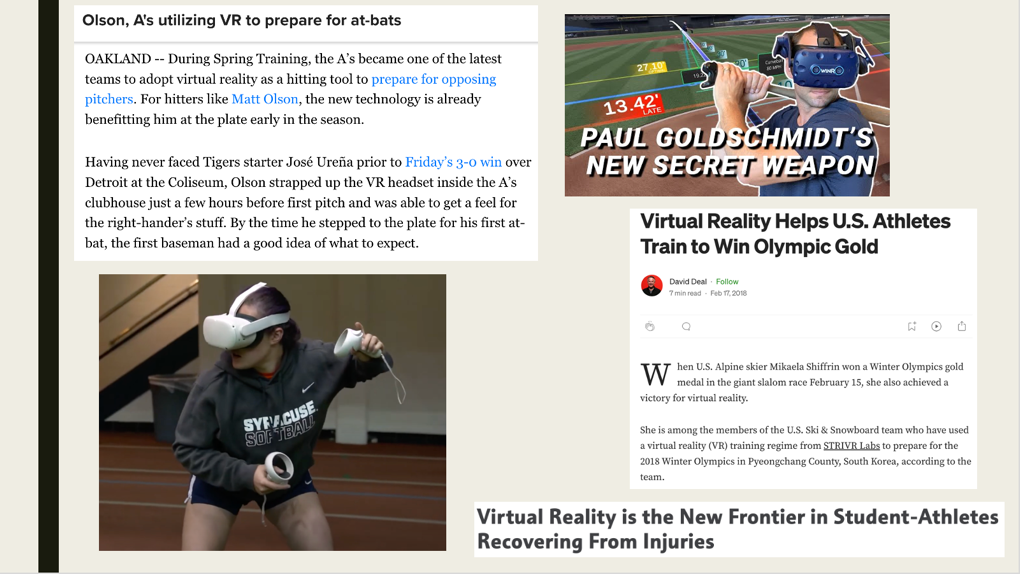

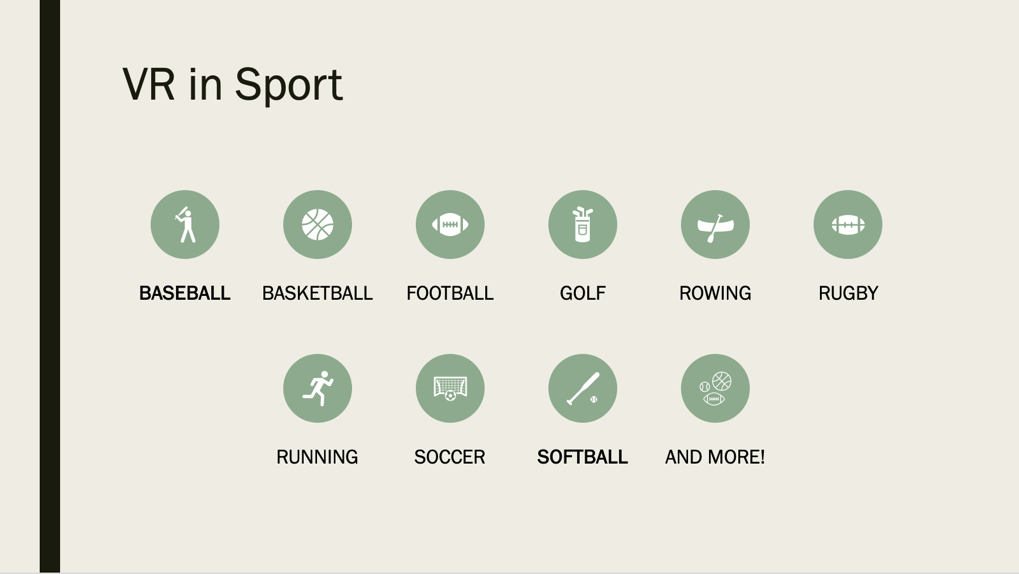


7

6

5

References

1. Harris ME, Mills RJ, Fawson C, Johnson JJ. Examining the impact of training in the unified theory of acceptance and use of technology. J Comput Inf Syst. 2018;58(3):221-33.

2. Lewellen JA, Baker E, Giacobbi PR, Jr. Athlete perceptions of virtual reality and barriers to its use in sport: A qualitative examination. PLoS One. 2025;20(4):e0261378.

3. Davis FD. Perceived usefulness, perceived ease of use, and user acceptance of information technology. MIS Q. 1989;13(3):319-40.

4. Carnegie K, Rhee T. Reducing visual discomfort with HMDs using dynamic depth of field. IEEE Comput Graph Appl. 2015;35(5):34-41.

5. Sepich NC, Jasper A, Fieffer S, Gilbert SB, Dorneich MC, Kelly JW. The impact of task workload on cybersickness. Front Virtual Real. 2022;3.

6. Woo YS, Jang K-M, Nam SG, Kwon M, Lim HK. Recovery time from VR sickness due to susceptibility: Objective and quantitative evaluation using electroencephalography. Heliyon. 2023;9(4):e14792.

7. Mascret N, Montagne G, Devrièse-Sence A, Vu A, Kulpa R. Acceptance by athletes of a virtual reality head-mounted display intended to enhance sport performance. Psychol Sport Exerc. 2022;61.
