# Supplementary material for: Assessing the feasibility of the Virtual Reality Education and Acceptance Protocol among baseball and softball players
Source: PLoS One. 2025 Nov 25;20(11):e0337537. doi: 10.1371/journal.pone.0337537 (PMC12646411; doi:10.1371/journal.pone.0337537)
Supplement: Attachment — Submitted filename: Response to Reviewers.docx [file pone.0337537.s003.docx]

**Manuscript PONE-D-25-29500**

**Response to Reviewers**

Dear Dr. Fransen and Reviewers,

Thank you for your feedback on our manuscript. We have taken all comments into consideration and provided our responses in blue below. Additionally, the changes are highlighted in the Revised Manuscript with Track Changes file.

Please also note that Microsoft Word has a glitch that changes the line numbers when Track Changes is set to “All Markup,” and that our line numbers below reflect the accurate continuous line numbers in the Manuscript file with no track changes, or when the track changes function is set to “Simple Markup” in the track changes file.

Best,

Jarad A. Lewellen and Peter R. Giacobbi, Jr.

**Journal requirements:**

When submitting your revision, we need you to address these additional requirements.

1. Please ensure that your manuscript meets PLOS ONE's style requirements, including those for file naming. The PLOS ONE style templates can be found at

<https://journals.plos.org/plosone/s/file?id=wjVg/PLOSOne_formatting_sample_main_body.pdf> and [https://journals.plos.org/plosone/s/file?id=ba62/PLOSOne_formatting_sample_title_authors_affiliations.pdf](https://track.editorialmanager.com/CL0/https:%2F%2Fjournals.plos.org%2Fplosone%2Fs%2Ffile%3Fid=ba62%2FPLOSOne_formatting_sample_title_authors_affiliations.pdf/1/010f0198a897c187-fb896684-5554-4188-93e9-2564cf69f131-000000/Q-4ZnxmsyjiPgLcaJynZkdPPPgvp6Ucw-EEDpRQKoR0=224)

**Author Response:** Thank you for bringing this to our attention. We have ensured that our revised submission meets the PLOS ONE style and file naming requirements.

2. Thank you for stating in your Funding Statement:

[This study was funded by the Stitzel Graduate Student Support Endowment awarded to the first author (JAL) at West Virginia University. The funders had no role in study design, data collection and analysis, decision to publish, or preparation of the manuscript.].

Please provide an amended statement that declares *all* the funding or sources of support (whether external or internal to your organization) received during this study, as detailed online in our guide for authors at <http://journals.plos.org/plosone/s/submit-now>.  Please also include the statement “There was no additional external funding received for this study.” in your updated Funding Statement.

Please include your amended Funding Statement within your cover letter. We will change the online submission form on your behalf.

**Author Response:** Thank you for bringing this to our attention. We have amended the funding statement as noted (please see above in cover letter).

3. Please include your full ethics statement in the ‘Methods’ section of your manuscript file. In your statement, please include the full name of the IRB or ethics committee who approved or waived your study, as well as whether or not you obtained informed written or verbal consent. If consent was waived for your study, please include this information in your statement as well.

**Author Response:** Thank you for this comment. Our Methods section begins with the following ethics statement: “This study was conducted after approval was obtained from the West Virginia University Institutional Review Board (Protocol #2410050857). All participants provided written informed consent before participating in the study.” To the best of our knowledge, this includes the required information noted in the comment. Please advise if there is still information missing.

If the reviewer comments include a recommendation to cite specific previously published works, please review and evaluate these publications to determine whether they are relevant and should be cited. There is no requirement to cite these works unless the editor has indicated otherwise.

Additional Editor Comments:

Dear authors
Our reviewers have recommended that significant revisions should be made before your manuscript can be considered further. Please consider their recommendations and address them. I will then make a consideration about whether these comments were addressed sufficiently before sending it back out for a new round of revisions.
Sincerely
Job

[Note: HTML markup is below. Please do not edit.]

Reviewers' comments:

Reviewer's Responses to Questions

**Comments to the Author**

1. Is the manuscript technically sound, and do the data support the conclusions?

The manuscript must describe a technically sound piece of scientific research with data that supports the conclusions. Experiments must have been conducted rigorously, with appropriate controls, replication, and sample sizes. The conclusions must be drawn appropriately based on the data presented.

Reviewer #1: Partly

Reviewer #2: Yes

2. Has the statistical analysis been performed appropriately and rigorously?

Reviewer #1: No

Reviewer #2: Yes

3. Have the authors made all data underlying the findings in their manuscript fully available?

The [PLOS Data policy](http://track.editorialmanager.com/CL0/http:%2F%2Fwww.plosone.org%2Fstatic%2Fpolicies.action%23sharing/1/010f0198a897c187-fb896684-5554-4188-93e9-2564cf69f131-000000/k69ext5q1v1F-cUDWrD4Jti7QXwxgypuf40_SJTZzQw=224) requires authors to make all data underlying the findings described in their manuscript fully available without restriction, with rare exception (please refer to the Data Availability Statement in the manuscript PDF file). The data should be provided as part of the manuscript or its supporting information, or deposited to a public repository. For example, in addition to summary statistics, the data points behind means, medians and variance measures should be available. If there are restrictions on publicly sharing data—e.g. participant privacy or use of data from a third party—those must be specified.

Reviewer #1: Yes

Reviewer #2: Yes

4. Is the manuscript presented in an intelligible fashion and written in standard English?

PLOS ONE does not copyedit accepted manuscripts, so the language in submitted articles must be clear, correct, and unambiguous. Any typographical or grammatical errors should be corrected at revision, so please note any specific errors here.

Reviewer #1: Yes

Reviewer #2: Yes

5. Review Comments to the Author

Please use the space provided to explain your answers to the questions above. You may also include additional comments for the author, including concerns about dual publication, research ethics, or publication ethics. (Please upload your review as an attachment if it exceeds 20,000 characters)

Reviewer #1: The authors have submitted an overall well-written paper exploring the feasibility and acceptability of VR training in baseball and softball. While this paper has some positives, I have some concerns that need to be addressed prior to being considered for publication.

**Author Response:** Thank you for your feedback! We have worked to address you commends that have undoubtedly strengthened our paper.

General comments:
- The introduction is well-written for the most part, but needs more work building on past research, differentiating between different types of extended reality and where VR fits in, and accuracy of cited studies. In addition to this, the rationale for the study is somewhat there, but needs to be clearer as it feeds into the aim.

**Author Response:** Thank you for your comments. We have addressed your comments below and feel that we have incorporated more recent research, provided an extended discussion on the XR spectrum, provided clarification on our cited studies, and expanded the explanation of the rationale.

- It is currently not clear what the Virtual Reality Training Protocol for Sport (VRTPS) is. Apologies if there is confusion, but have you created this protocol? What were the steps taken to create this protocol? Or was this protocol adapted from previous research - if so, it has not been covered in the introduction and needs to be. This is a significant current limitation of the study. How confident are you that this should be called Virtual Reality Training Protocol for Sport, when it is focusing on baseball/softball specifically (very specific types of sport)? I would caution against this title.

**Author Response:** Thank you for you comments and questions. We did create this protocol, and we have attempted to make that clearer in the manuscript, specifically in the Virtual Reality Education and Acceptance Protocol subsection (beginning on Line 245). We understand your suggestion that we include the development of the protocol in the introduction. However, because it has not been empirically tested prior to this study and the primary aim is to assess its feasibility, we feel it fits best in the methods, though we did provide more context and detail at the end of the introduction (Lines 204-209). After considering your feedback, we also changed the name of the protocol to more accurately reflect its purpose. We chose to keep the title general, as we intend for the VREAP to be adapted to various domains in future research and application, though we specified that it was adapted to use with baseball and softball players in the title and throughout the manuscript. We have also included more research from other domains to provide more justification for the general name of the protocol.

- It is not clear why statistical analyses were not used to understand differences. I understand there is a small sample size, but please elaborate more and whether there are non-parametric (or other) statistics you could possibly use.

**Author Response:** Thank you for this comment. After considering reviewer feedback, we have conducted nonparametric tests (i.e., Wilcoxon signed-ranked test) on the AVRTS and VRSQ pre- and post-test means and incorporated the outputs into the results and discussion sections as appropriate. Because the subsection of the results that focused on the VRSQ and AVRTS now includes these analyses (i.e., more than just descriptive trends), we have renamed the section “Cybersickness and attitudes toward VR” (Line 486).

Minor comments:
Abstract
L19 - I would suggest adding cost here also.

**Author Response:** Thank you for your suggestion. We have added cost to the end of this sentence (see Line 30).

Introduction
L54-55 - This study was not a training study, please amend.

**Author Response:** Thank you for bringing that to our attention. We have amended the sentence to remove the suggestion that VR was used for training in this study (see Line 79).

L56 - Although the article title suggest VR, Page and colleagues used 360-video, not VR. Please amend, and I recommend providing a greater overview of VR/Extended Reality (XR) earlier in the introduction to discriminate between VR, 360-video, MR, AR.

**Author Response:** Thank you for this suggestion. We have included an overview of XR, VR, AR, and MR early in the discussion (Lines 49-61). We have also noted the difference between animated VR and 360-video using a VR headset and specified that the Page et al. study used 360-video (Line 82).
L80-83 - There are several recent reviews that have explored VR in sport (training). I recommend engaging more with this literature to support your statements here.

**Author Response:** Thank you for this suggestion. We have included findings from two recent reviews (Cariati et al., 2025; Witte et al., 2025) to support our statements (Lines 106-115).

L85 - What evidence? No evidence is cited.

**Author Response:** Thank you for the note. We have reworded the sentence for clarity and included a recent study that demonstrates a lack of VR use in collegiate sport (Lines 116-119).

L86-89 - This is a very relevant study to your current study, and I believe it warrants further discussion than a single sentence.

**Author Response:** Thank you for this suggestion. We have included a paragraph with more detail on this study and its findings (Lines 123-137).

L94 - Is this 80% context-specific? This is a strong statement, and needs further elaboration. If 80% of all VR users experienced cybersickness, very few people would use it.

**Author Response:** Thank you for this note. We decided to remove this sentence from the manuscript as we felt the previous sentence and reference (Lewellen et al., 2025) was sufficient evidence that cybersickness is a barrier. Additionally, after a more careful review of the cybersickness literature, it is difficult to ascertain an accurate percentage of cybersickness likelihood, as there is great variation between studies, and many of them are intentionally eliciting symptoms, which does not accurately reflect the possibility of cybersickness when symptoms are not induced.

L130-134 - The rationale here seems very short. I recommend expanding on this more to help set up the aims of the study.

**Author Response:** Thank you for this recommendation. We elaborated on the rationale and purpose of the study in Lines 203-214.

I recommend including this study, or justify the choice to exclude this: Wilkins, L. (2024). A framework for using virtual reality to enhance psychological performance in baseball. Journal of Sport Psychology in Action, 1-16.

**Author Response:** Thank you for this recommendation. We chose not to include this study in the introduction because it was not experimental and thus did not support our rationale and aims. However, we agree it is an important study, especially as it relates to the applied implications of our study, so we included it in our discussion of future directions (Lines 666-677).

Methods
L186 - Please cite multiple studies as you have said 'similar feasibility studies'/

**Author Response:** Thank you for this note. After careful review, we felt the systematic review that we originally referenced was not an accurate enough comparison to our study and was not necessary to reference. Instead, we felt that concluding this paragraph with the adherence rate from Ross-Stewart et al.’s (2018) study created better flow. We also changed “engagement” to “adherence” in Line 322 to accurately reflect the language used in Ross-Stewart et al. (2018).

L223 - Please cite the previous literature here - this is vital.

**Author Response:** Thank you for this suggestion. We referenced the relevant previous literature throughout this section as each stage was discussed. We added a sentence explaining this for clarity (Line 248). We also added additional references to support Stages 1 and 3 (Lines 260; 284).

L251-252 - Why did you choose to use the controllers only and not the bat? I am surprised this was not included when testing the acceptability of high level participants. This is a design issue that would have overcome some of the comments raised in the Results (though I think these are important to raise)

**Author Response:** Thank you for the question. There were two primary reasons we did use bats. 1) We had budget constraints and could not afford to purchase bats or bat attachments for this study. Given that participants did not have to be actively participating in the sport, we also could not rely on them to bring their own bats. 2) Because participants were run in groups, there were four participants using VR simultaneously in the same room. Given the space, we did not want to risk potential injury to participants with swinging, and possibly accidentally letting go of, the bats. Because we understand this could be an important limitation, we also added this context in the Discussion (Lines 627-628).

Results
L303-325 - Apologies if incorrect for feasibility studies, but I feel this would sit better in the Methods? If this is standard practice for feasibility practices, happy for it to remain here.

**Author Response:** Thank you for the suggestion. While we understand why it might flow better in the Methods, it is standard practice to include this in the results because successful/unsuccessful recruitment methods are a result themselves in a feasibility study. Please see similarly designed feasibility studies below as examples:

- Garcia JM, Shurack R, Leahy N, Brazendale K, Lee E, Lawrence S. Feasibility of a remote-based nutrition education and culinary skills program for young adults with Autism Spectrum Disorder. J Nutr Educ Behav. 2023;55(3):215-23.
- Sandgren SS, Haycraft E, Arcelus J, Plateau CR. Evaluating a motivational and psycho-educational self-help intervention for athletes with mild eating disorder symptoms: A mixed methods feasibility study. Eur Eat Disord Rev. 2022;30(3):250-66.

L384 - Why have no statistical analyses been done here? I am not sure you can say 'increased minimally' - this would be no significant difference.

**Author Response:** Thank you for this question. Initially, we did not include parametric tests because the primary focus of the study was to assess feasibility. However, as noted above, after considering reviewer feedback, we have conducted nonparametric tests (i.e., Wilcoxon signed-rank test) on the AVRTS and VRSQ pre- and post-test means and incorporated the outputs into the results and discussion sections as appropriate.

Discussion
L418 - Please clarify that this was through interview responses.

**Author Response:** Thank you for the suggestion. We included this in the sentence (Line 537). We also included that statistical analyses suggest potential of the VREAP, as results from the surveys and the Wilcoxon signed-rank test indicated high acceptance and low cybersickness.

L436 - Revise wording to start this sentence.

**Author Response:** Thank you for bringing this to our attention. The sentence has been revised.

L438-44 - I was very surprised these studies were not included in the introduction. Please include these and a deeper discussion to help provide background for your study.

**Author Response:** Thank you for the suggestion. We have included a deeper discussion of these studies in the introduction (Lines 138-153).

L452-456 - Do you think that running the data collection in groups (and the conversational nature among these groups) may have influenced the results?

**Author Response:** Thank you for this question. It is possible groups could have affected the results. We feel that we provided an explanation of why participants were run in groups (Lines 356-257), as well as addressed this possibility in the limitations subsection (Lines 645-648). We also added “and thus the results” in Line 646 for added clarity.

L493 - I agree that this limits the generalisability - I therefore caution against a general term such as VRTPS if you are acknowledging that it might not be generalised to other sports. As you say in L539 - "future research should confirm these findings and examine the efficacy of the VRTPS for various sports"

**Author Response:** Thank you for this comment. We understand that suggesting the protocol is “for sport” can initially be misleading. As noted above, we changed the name of the protocol but chose to make the title more general, as we intend for the VREAP to be adapted to various domains in future research and application. We believe there is space for the title to be more general because the purpose of the VREAP is for education and acceptance, not impacting performance, which would demand more specificity. Rather, it was designed with general theories and research in mind that apply to many domains (e.g., Technology Acceptance Model, cybersickness), so we believe it is acceptable to have a general title. However, we specified that it was adapted to use with baseball and softball players in the title and throughout the manuscript. We also provided this context in the section noted in this comment (Lines 832-838).

L506 - Why did you not provide gender comparisons? This should be an easy and important comparison to run.

**Author Response:** Thank you for this question. As noted above, we did not initially include gender comparisons because the primary focus of the study was to assess feasibility. However, after considering reviewer feedback, and because we have a particularly small sample size and an uneven distribution between genders, we conducted two Wilcoxon signed-ranked tests to examine differences over time for each gender independently rather than comparing their changes to each other. We provide our results and interpretation in the corresponding sections.

Thank you for the opportunity to review and best of luck.

**Author Response:** Thank you, and we appreciate you taking the time to review!

Reviewer #2: To enhance flow I would prefer the Virtual Reality Training Protocol for Sport (VRTPS) section to be positioned before the questionnaire section.

**Author Response:** Thank you for the suggestion. We have repositioned the VREAP (formerly VRTPS) subsection ahead of the Measures subsection to enhance flow.

Can you clarify the experience level of the four coders please.

**Author Response:** Thank you for the suggestion. We have provided more detail on the coders’ experience level (Lines 392-395).

Can you provide more detail on the content analysis process.

**Author Response:** Thank you for the suggestion. We have included more detail on the content analysis of the observation notes (Lines 398-401).

Results are logical, well-presented and appropriate. Strong discussion and conclusion.

**Author Response:** Thank you for your feedback and for taking the time to review!
