# Supplementary material for: Assessing the feasibility of the Virtual Reality Education and Acceptance Protocol among baseball and softball players
Source: PLoS One. 2025 Nov 25;20(11):e0337537. doi: 10.1371/journal.pone.0337537 (PMC12646411; doi:10.1371/journal.pone.0337537)
Supplement: Attachment — Submitted filename: Response_to_Reviewers_auresp_2.docx [file pone.0337537.s004.docx]

**Manuscript PONE-D-25-29500**

**Response to Reviewers**

Dear Dr. Fransen and Reviewers,

Thank you for your additional feedback on our manuscript. We have taken all comments into consideration and provided our responses in blue below. Additionally, the changes are highlighted in the Revised Manuscript with Track Changes file. In this file, Per Reviewer #1’s request, we have also marked the additional changes to the manuscript in red font to make it easier to identify where changes have been made.

Please also note that Microsoft Word has a glitch that changes the line numbers when Track Changes is set to “All Markup,” and that our line numbers below reflect the accurate continuous line numbers in the Manuscript file with no track changes, or when the track changes function is set to “Simple Markup” in the track changes file.

Best,

Jarad A. Lewellen and Peter R. Giacobbi, Jr.

**Journal Requirements:**

If the reviewer comments include a recommendation to cite specific previously published works, please review and evaluate these publications to determine whether they are relevant and should be cited. There is no requirement to cite these works unless the editor has indicated otherwise.

Please review your reference list to ensure that it is complete and correct. If you have cited papers that have been retracted, please include the rationale for doing so in the manuscript text, or remove these references and replace them with relevant current references. Any changes to the reference list should be mentioned in the rebuttal letter that accompanies your revised manuscript. If you need to cite a retracted article, indicate the article’s retracted status in the References list and also include a citation and full reference for the retraction notice.

**Author Response:** Thank you for these comments. To the best of our knowledge, none of our cited papers have been retracted. We have also reviewed our reference list to ensure accuracy (e.g., reviewed formatting and included DOIs).

Dear authors
Reviewer one has some minor changes they would like to see being addressed before we can proceed.

[Note: HTML markup is below. Please do not edit.]

Reviewers' comments:

Reviewer's Responses to Questions

**Comments to the Author**

1. If the authors have adequately addressed your comments raised in a previous round of review and you feel that this manuscript is now acceptable for publication, you may indicate that here to bypass the “Comments to the Author” section, enter your conflict of interest statement in the “Confidential to Editor” section, and submit your "Accept" recommendation.

Reviewer #1: (No Response)

Reviewer #2: All comments have been addressed

2. Is the manuscript technically sound, and do the data support the conclusions?

The manuscript must describe a technically sound piece of scientific research with data that supports the conclusions. Experiments must have been conducted rigorously, with appropriate controls, replication, and sample sizes. The conclusions must be drawn appropriately based on the data presented.

Reviewer #1: Yes

Reviewer #2: Yes

3. Has the statistical analysis been performed appropriately and rigorously?

Reviewer #1: Yes

Reviewer #2: Yes

4. Have the authors made all data underlying the findings in their manuscript fully available?

The [PLOS Data policy](http://www.plosone.org/static/policies.action#sharing) requires authors to make all data underlying the findings described in their manuscript fully available without restriction, with rare exception (please refer to the Data Availability Statement in the manuscript PDF file). The data should be provided as part of the manuscript or its supporting information, or deposited to a public repository. For example, in addition to summary statistics, the data points behind means, medians and variance measures should be available. If there are restrictions on publicly sharing data—e.g. participant privacy or use of data from a third party—those must be specified.

Reviewer #1: Yes

Reviewer #2: Yes

5. Is the manuscript presented in an intelligible fashion and written in standard English?

PLOS ONE does not copyedit accepted manuscripts, so the language in submitted articles must be clear, correct, and unambiguous. Any typographical or grammatical errors should be corrected at revision, so please note any specific errors here.

Reviewer #1: Yes

Reviewer #2: Yes

6. Review Comments to the Author

Please use the space provided to explain your answers to the questions above. You may also include additional comments for the author, including concerns about dual publication, research ethics, or publication ethics. (Please upload your review as an attachment if it exceeds 20,000 characters)

Reviewer #1: Well done to the authors for their thorough revisions of this manuscript. I appreciate that a long list of revisions can be a challenge, but the authors have done a thorough job. One recommendation I will make to the authors is to have any changes made to the revised manuscript in red font. This would make it easier to review and clearly identify where changed were made.

**Author Response:** Thank you for your kind comments and suggestion. We have marked all changes to this version of revisions in red font (in the Revised Manuscript with Track Changes file) to make the changes easier to identify.

I appreciate the authors' changes to the title and think that this is a better title for the study.
My main suggestion is for parts of the discussion to be expanded to really tie together the paper's findings. The main parts to focus on are the paragraphs from Line 552 to Line 576. I think you could draw on research in sport using VR (and other XR technologies) to highlight whether these have been more game like, enjoyment, difficult to use. You could also consider bringing in work from other domains such as education, medical training.

**Author Response:** Thank you for these suggestions. We have expanded on the content in these paragraphs to tie our findings together with other research. Specifically, we included references on game-like and realistic experiences among exercise populations, impacts of perceived usefulness and enjoyment on demand among coaches, and potential impact of eyeglass use and HMD discomfort on VR experience and acceptance (Lines 557-589).
Well done again and best of luck. I look forward to hopefully applying this protocol in my own XR research.

**Author Response:** Thank you for this comment and for taking the taking the time to review our manuscript!

Reviewer #2: The study is interesting. The use of 360 and VR is still intermittent across sports, so the findings will add value to the discussions in the area.

**Author Response:** Thank you for this feedback and for taking the time to review our manuscript!
